# Psychometric properties of the Italian version of the Parent Experience of Assessment Scale

**DOI:** 10.3389/fpsyg.2023.1271713

**Published:** 2024-02-01

**Authors:** Filippo Aschieri, Sara Brasili, Anna Cavallini, Giulia Cera

**Affiliations:** ^1^Department of Psychology, Università Cattolica del Sacro Cuore, Milan, Italy; ^2^Department of Child Neuropsychiatry, Fondazione Don Gnocchi, Milan, Italy

**Keywords:** child assessment, therapeutic assessment, parent satisfaction, PEAS, psychometric properties, confirmatory factor analysis, structural equation modeling

## Abstract

This paper describes the psychometric properties of the Italian version of the Parent Experience of Assessment Scale. Overall, 185 participants took part in the study. Confirmatory factor analysis and structural equation modeling tested the scale structure and its relationship with clients’ satisfaction. Reliability and multivariate analysis of variance measured the factors’ consistency and the differences among different typologies of assessment. Results replicated the original five factors structure of the scale (Parent-Assessor Relationship and Collaboration; New Understanding of the Child; Child-Assessor Relationship; Systemic Awareness; Negative Feelings). Full scale and individual factors’ reliability ranged from high to excellent. Structural equation modeling showed that Parent-Assessor Relationship and Collaboration and New Understanding of the Child factors had the strongest direct effects on parents’ General Satisfaction, measured by the Client Satisfaction Questionnaire. A multivariate analysis of variance showed that the type of assessment, the children’s age and the way the scale was completed impacted on the outcomes of the QUEVA-G. Results suggest that the Italian version of the Parent Experience of Assessment Scale is a valid and reliable tool for assessing parents’ experience of their child’s assessment.

## Introduction

Customers’ satisfaction, opinions, and perceptions are considered crucial indicators to evaluate the effectiveness and quality of service and to define its benefits and possible improvements (Lebow, 1983; Farmer and Brazeal, 1998; McMurtry and Hudson, 2000). However, until a few years ago, the practice of assessing clients’ satisfaction was exclusively based on the practitioner’s experience or scales with unknown psychometric properties (Young et al., 1995). Hence, in the past decades, there has been an increased interest in the development of valid and reliable measurement instruments to assess customers’ satisfaction in multiple contexts. To date, the Client Satisfaction Questionnaire (CSQ; Larsen et al., 1979), available in 5 different versions (namely, CSQ-3, CSQ-4, CSQ-8, CSQ-18A, and CSQ-18B), is the most commonly used single-factor measure of satisfaction.

Most consumers’ satisfaction research has been focusing on medical and healthcare services for adult patients and clients, while very few studies have been dedicated to childcare services. In this field, satisfaction with children’s mental health services is measured through their parents’ reports. Many studies carried out so far on parental satisfaction with childcare services have been focused on mental health treatment (Byalin, 1993; Young et al., 1995; Brannan et al., 1996; Godley et al., 1998; Martin et al., 2003; Riley et al., 2005) and selected populations, for example, severely emotionally disturbed children (Rouse et al., 1994), disabled children (Clare and Pistrang, 1995), or children with chronic health problems (King et al., 1996). As a result, information on parental satisfaction with their child’s assessment services is still limited. This lack of research is potentially problematic because parents’ satisfaction, as an outcome of the assessment process, is highly relevant to promote family engagement in treatment recommendations.

In Italy, specifically, public mental health services face a significant influx of requests and lengthy waitlists. The more effectively assessors can engage families in the assessment of their children, the greater the likelihood that these families will effectively utilize the long-anticipated assessment results.

To fill the gap in the literature and provide a specific measure of parents’ experience with children’s psychological assessment services, Austin (2011) developed the Parent Experience of Assessment Scale (PEAS, Austin, 2011), a 24-item scale that measures five factors: Parent–Assessor Relationship and Collaboration (PARC), New Understanding of the Child (NUC), Child–Assessor Relationship (CAR), Systemic Awareness (SA), and Negative Feelings (NF). The scale exhibited appropriate internal consistency reliability (Cronbach’s alpha from 0.76 to 0.88). Additionally, evidence of convergent construct validity has been provided through significant two-tailed Pearson correlations between the revised PEAS subscales and the CSQ-8 scores (Pearson’s *r* between 0.20 and 0.64; *p* < 0.05).

In their study (Austin et al., 2016), the authors compared three models: (1) a first-order model with five correlated factors; (2) a second-order model, in which it was assumed that a hierarchical factor, called “General Satisfaction,” could account for the covariance of the PEAS subscales; and (3) another second-order model in which the previous General Satisfaction factor was replaced by the PARC factor. This final model showed the best fit for the data. Austin et al. (2016), while testing different factor structures through CFA, emphasized a pragmatic rationale. Indeed, the PARC factor was used as a second-order factor based on the empirically assessed covariances among it and the other first-order factors, as well as on its 0.96 covariance with the General Satisfaction factor of the previous model.

Also, the authors’ findings may provide an overly positive picture of the scale fit and its ability to predict parental satisfaction. Indeed, the authors added modification indices between errors pertaining to items from different factors: item 2 (PARC) and 14 (CAR), 9 (NUC) and 14 (CAR), 15 (CAR) and 16 (SA), 4 (PARC) and 12 (NUC), and 7 (PARC) and 16 (SA). Furthermore, while employing a structural equation model (SEM) to investigate which of the PEAS subscales were predictive of the General Satisfaction factor given by the CSQ-8, they represented this domain as an observed variable rather than an estimated variable.

In our study, on the contrary, we aim to maintain separation between a theory-driven CFA and a data-driven SEM (Sorgente et al., 2023). Our confirmatory factor analysis compared two models: one in which the five factors were considered as correlated factors of the measure of parents’ assessment experience, and one in which the five first-order factors had an overarching second-order factor accounting for their covariances. In the SEM, we tested which configuration of the QUEVA-G’s factors accounts best for parents’ satisfaction measured through the CSQ-8 items.

In Italy, there has been no research on any of the broadband scales to measure clients’ satisfaction, let alone those dedicated to children’s psychological services. Hence, this study aimed to translate and validate the Parent Experience of Assessment Scale (PEAS; Austin, 2011) in an Italian sample of parents. The development of an Italian scale for measuring parental satisfaction with children’s assessment would allow us to (1) evaluate the quality of the psychological assessment services provided to clients; (2) collect valuable feedback about how to improve the delivery of the services; and (3) promote research on the effects of delivering psychological assessment to children and their families using more traditional or collaborative/therapeutic models (Tharinger et al., 2022).

### Aim of the project

This study has four aims. The first aim is to investigate the structure of the five-factor model of the Italian version of PEAS (Questionario sull’Esperienza della Valutazione dei Genitori, QUEVA-G; (Appendix A)). The second aim is to evaluate the QUEVA-G’s reliability. The third aim is to predict general satisfaction for children’s psychological assessment (measured through the CSQ-8) through the QUEVA-G. Finally, the fourth aim is to explore, without any a-priory hypotheses, the effects of the administration (paper or online), children’s features (gender and age), and type of assessment on the parents’ experience of their child’s assessment.

## Methods

### Sites

In our study, we collected data through both paper (*n* = 35) and online questionnaires (*n* = 150). Paper questionnaires were distributed at several facilities in the northern region of Italy, particularly in Milan and its surrounding areas. Specifically, two facilities provided the majority of paper-based data: a private practice specializing in neuropsychological assessments (*n* = 11) and a private psychological and neuropsychological clinic in Milan (*n* = 24). The facilities participating in data collection responded affirmatively to our request for collaboration in this research study. Initially, the invitation was extended to the network of public mental health services in Milan as well as to several private centers. One of the co-authors, Anna Cavallini, oversaw the administration of the paper version of the questionnaire. The staff of the two facilities administered the questionnaires to parents at the end of the assessment. Once parents responded to the questionnaires, they left them, anonymously, in a box in which all questionnaires were collected.

The online questionnaires were administered through Qualtrics and distributed via social networks. The links to the questionnaires were distributed in self-help groups for parents of children with psychological diagnoses or in self-help groups for parents. Data collection was anonymous.

### Participants

We recruited parents whose children completed a psychological evaluation less than a year before the scale’s administration to ensure that the memory of the assessment was still vivid. For example, children were assessed for either emotional–behavioral problems, cognitive–neurodevelopmental issues, or the co-occurrence of both types of problems. All questionnaires were completed after the last session of the assessment. There were no exclusion criteria in terms of children’s diagnosis, children’s level of functioning, or the type of assessment completed.

Altogether, 212 respondents participated in the study. Twenty-three participants opened the questionnaire link but did not provide any response. Among the remaining 189 participants, three individuals were excluded because their child’s age at the time of assessment was outside the prescribed range of 4–18 years. Ultimately, one additional case was excluded due to random responses. One hundred eighty-five protocols were included in the analyses (Table 1).

**Table 1:** Descriptive statistics of participants.

Variables	*n*	%
Format of administration		
Online	150	81.1
Paper form	35	18.9
Gender of the parent		
Male	11	5.9
Female	174	94.1
Kinship		
Biological parents	176	95.7
Adoptive parents	5	2.7
Foster parents	1	0.5
Other first-degree relatives	2	1.1
Gender of the child		
Male	127	68.6
Female	58	31.4
Age range		
4–11	122	65.95
12–18	63	34.05
*M* = 10.41 *SD* = 3.4 min = 4 max = 18		
Origin of the family		
Italy	178	96.2
Africa	1	0.5
Asia	2	1.1
Eastern Europe	3	1.6
Latin America	1	0.5
Type of assessment		
Cognitive and neurodevelopmental	116	62.7
Emotional and behavioral	15	8.1
Cognitive and emotional (mixed)	20	10.8
Unidentified	34	18.4

*n* = 185.

Most of the respondents to the questionnaires in our research were female respondents (*n* = 174); only a small percentage of the total sample were male respondents (*n* = 11). In almost all cases, respondents were biological parents (*n* = 176), but in our sample, there were also adoptive parents (*n* = 5), foster parents (*n* = 1), and other first-degree relatives (*n* = 2). The majority of families were of Italian descent (*n* = 178); nevertheless, among the paper-based data collected at the two facilities, there were families hailing from Africa (*n* = 1), Asia (*n* = 2), Latin America (*n* = 1), and Eastern Europe (*n* = 3). Despite the different geographical origins, all participants were able to understand and answer the questions; prior to administering the questionnaire to the individuals from other countries, the research team ensured that their comprehension of the Italian language was adequate by asking the psychologists who had the opportunity to interact with the parents during their child’s evaluation process.

### Instruments

#### The Italian version of the Parent Experience of Assessment Scale (QUEVA-G)

The QUEVA-G consists of 24 items, rated using a 5-point Likert-type scoring system. The scale is composed of five factors. Parent–Assessor Relationship and Collaboration (7 items) includes the parents being informed about each step in the assessment process and having a positive, supportive, and empathetic relationship with the assessors (feeling the assessors were genuinely interested in helping, and feeling respected, liked, and listened to them). New Understanding of the Child (5 items) focuses on the chance that, at the end of the assessment, parents might know better how to deal with their child, understand his or her feelings and behaviors, and be provided with new and more effective parental skills. The Child–Assessor Relationship (4 items) investigates the parents’ perception of the relationship between their child and the assessors in terms of empathy, tuning, support, and understanding. Systemic Awareness (4 items) focuses on the possibility that parents may be able to recognize in a more systemic way their child’s problems and to understand that the whole family needs to change to help him or her. Negative Feelings (4 items) explores how much parents felt blamed, ashamed, or judged during the assessment. The scale was translated into Italian and back-translated into English prior to its administration, and the final version of the scale was approved by a bilingual author of the original study (S.E. Finn). Subsequently, to ensure its comprehensibility, the questionnaire was administered in a pilot study to a subset of families. Table 2 shows correlations among subscales.

**Table 2:** Correlations among the QUEVA-G subscales.

Subscale	1	2	3	4	5
1.Parent–Assessor Relationship and Collaboration	–				
2.New Understanding of the Child	0.539**	–			
3.Child–Assessor Relationship	0.641**	0.470**	–		
4.Systemic Awareness	0.037	0.145*	−0.290	–	
5.Negative Feelings_(R)	0.539**	0.312**	0.440**	−0.318**	–

*n* = 185. R, reverse scored.*p < 0.05. **p < 0.01.

#### The Client Satisfaction Questionnaire

The client satisfaction Questionnaire (CSQ-8; Larsen et al., 1979; Attkisson and Zwick, 1982). The CSQ-8 is a measure of clients’ general satisfaction and consists of 8 items using a 4-point Likert-type scale with four reverse-scored items (items 1, 3, 6, and 7). The Italian version of the CSQ-8 is protected by copyright, and its items cannot be publicly distributed. However, the scale can be obtained from Dr. Attkisson through appropriate permission. In our study, the CSQ-8 exhibited excellent reliability, as indicated by a Cronbach’s alpha coefficient of 0.97.

### Procedure

The study obtained the Catholic University of the Sacred Heart institutional review board approval (number of the practice: 42–23). Both paper and online questionnaires included the description of the study, the informed consent, and the two scales, i.e., the QUEVA-G and the CSQ-8.

### Analyses

Confirmatory Factor Analysis (CFA) and Structural Equation Modeling (SEM) were conducted with SPSS Amos version 29.0. The following parameters were used to evaluate the models: Chi-square (*X^2^*), degrees of freedom (*df*), discrepancy index (*X^2^/df*), value of p (*p*), comparative fit index (CFI), root mean square of approximation (RMSEA), standardized root mean square residual (SRMR), Tucker Lewis index (TLI), and Akaike information criterion (AIC). Discrepancy index (*X^2^/df*) values lower than 3 indicate a good fit of the model to the data (Kline, 2004). Comparative fit index (CFI) values above 0.95 indicate a good fit of the model to the data (Hu and Bentler, 1999; West et al., 2012). Root mean square of approximation (RMSEA) and standardized root mean square residual (SRMR) indicate good adaptability of the model to the data with values below 0.008 (Hu and Bentler, 1999). Tucker-Lewis index (TLI) indicates a good fit of the model to the data when above 0.90 (Byrne, 1994) or 0.95 (Hu and Bentler, 1999; West et al., 2012). Finally, regarding the Akaike information criterion (AIC), the best model is the one that explains the greatest amount of variability using the smallest number of independent variables; therefore, lower AIC values are preferred. If a model has an AIC lower by two units than another, then it can be considered significantly better (Wagenmakers and Farrell, 2004).

There were virtually no missing data for the 185 QUEVA-G protocols, with only 2 missing out of 4,440 individual item responses, for a total of 0.045% missing responses. These two missing data were estimated by calculating the mean of responses given to items belonging to the same subscale of QUEVA-G.

Correlation and SEM were run on a total of 177 individuals since 8 respondents did not complete the CSQ-8. Two missing answers in the CSQ-8 scale out of 1,416 individual item responses, for a total of 0.14% missing responses, were estimated by calculating the mean of responses given to the remaining items of the CSQ-8.

Other analyses (such as descriptive statistics, Cronbach’s alpha, and MANOVAs) were conducted using SPSS 27.0. Cronbach’s alpha has been estimated for each subscale and the total QUEVA-G questionnaire. A MANOVA was used to analyze the differences in the subscales among socio-demographics for child and parent respondents and among the type of assessment. Results were commented if alpha was below 0.05, and differences between groups were interpreted according to their effect size (Cohen, 1988).

## Results

### Analysis 1: scale factor structure

We tested the fit of the first-order model and a higher-order model as in Austin et al. (2016).

#### First-order model

**Figure 1:**
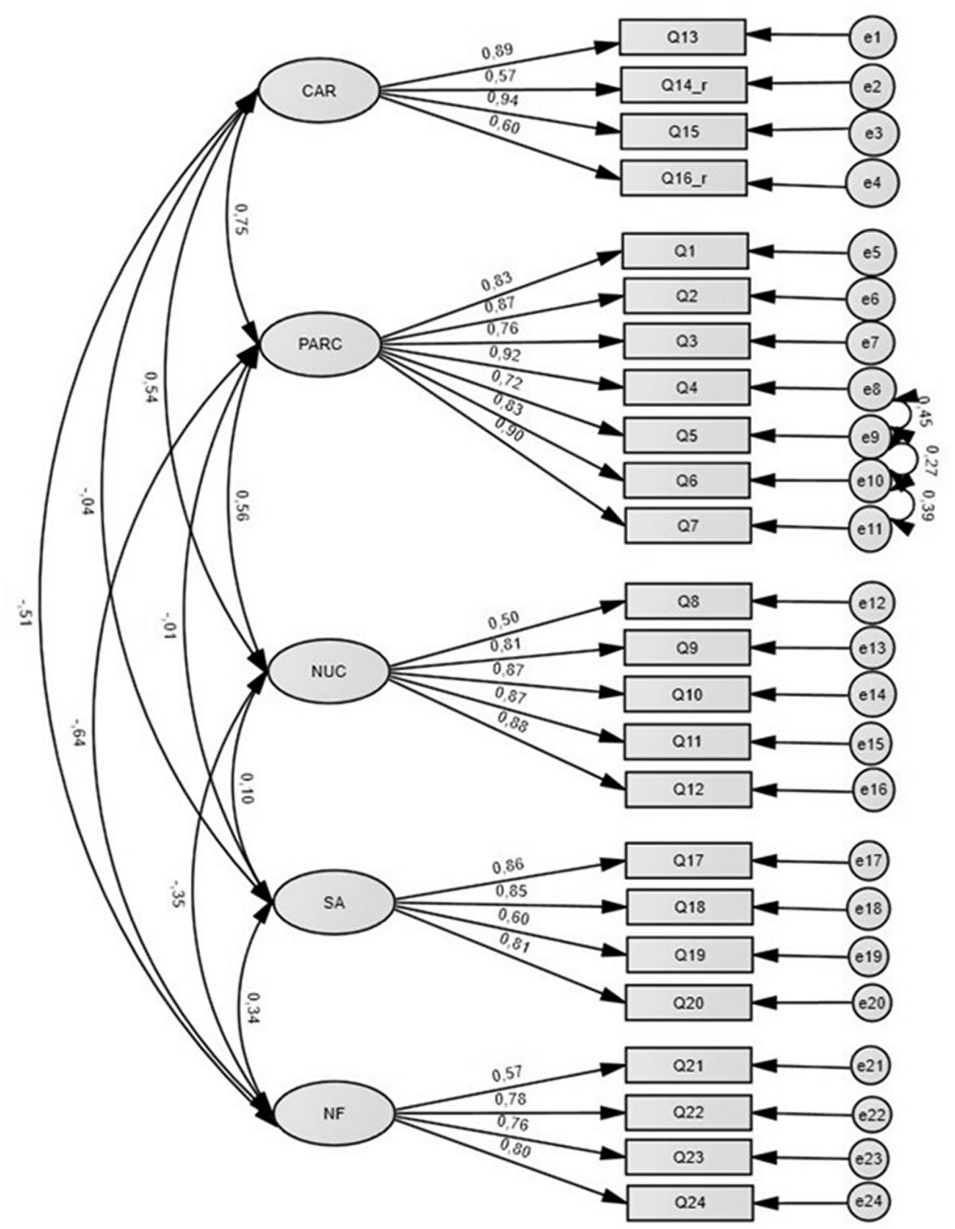
First-order CFA model and standardized coefficients (with modification indices).

In the first-order model (Figure 1), we assumed five correlated factors. Standardized loadings for all items were above 0.50. Modification indices suggested that we correlate error terms for items 4 and 5 (belonging to the factor “Parent–Assessor Relationship and Collaboration”), for items 6 and 7 (belonging to the factor “Parent–Assessor Relationship and Collaboration”), and for items 5 and 6 (belonging to the factor “Parent–Assessor Relationship and Collaboration”). Although *X^2^* for this model was statistically significant, all other fit indices suggested a good fit of the model to the data (Table 3). In our first-order model, significant covariances are observed only among four subscales, such as PARC, CAR, NUC, and NF. The highest covariances are between PARC and CAR (*r* = 0.75), NF (*r* = −0.64), and NUC (*r* = 0.56), similar to what was found by Austin et al. (2016). On the contrary, SA seems to be a relatively more independent dimension, being weakly correlated only with the NF subscale (*r* = 0.34). This suggests that in this sample, the more parents realize their personal implication in the child’s difficulties, the more likely it is that they will develop negative feelings in the assessment.

**Table 3:** First-order and second-order CFA model fit indices.

Model	*X^2^*	*df*	*p*	*X^2^*/*df*	TLI	CFI	RMSEA	95% CI for RMSEA	SRMR	AIC
First-order model	406.215	239	0.000	1.699	0.918	0.948	0.062	[0.051; 0.072]	0.0654	528.215
Second-order model	432.198	244	0.000	1.771	0.933	0.941	0.065	[0.055; 0.075]	0.0698	544.198

*X^2^*, Chi-square; df, degrees of freedom; *p*, *p* value; *X^2^*/*df*, discrepancy index; CFI, comparative fit index; RMSEA, root mean square of approximation; SRMR, standardized root mean square residual; TLI, Tucker-Lewis index; AIC, Akaike information criterion.

#### Second-order model

**Figure 2:**
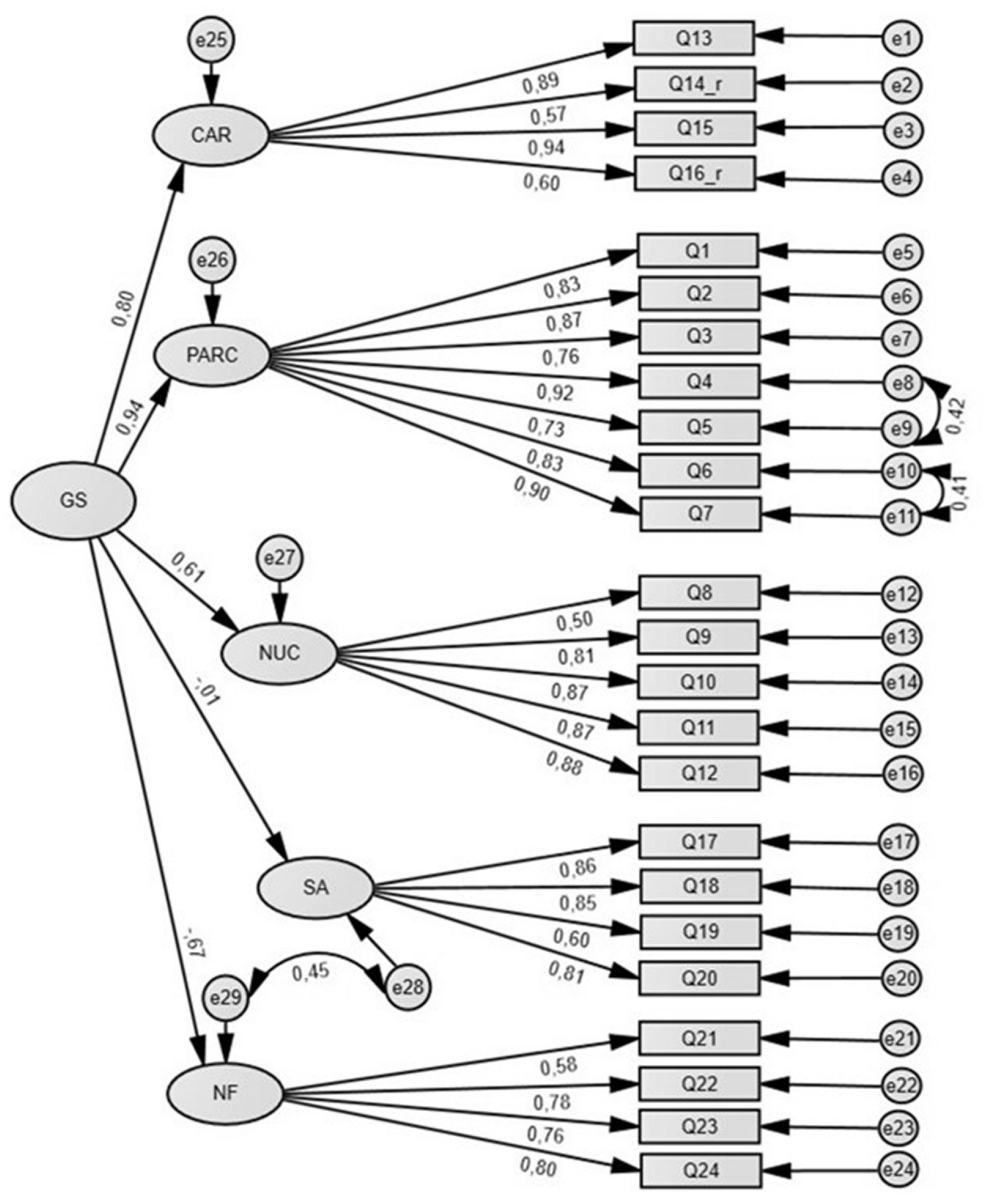
Second-order CFA model (with modification indices).

A second-order (hierarchical) model was also tested (Figure 2). We assumed that a hierarchical factor, called “General Satisfaction,” could explain other factors’ variances. Allowing for the covariance of the same error terms of items as in model 1, this model shows a good fit to the data. Although the two models both have a good fit, Table 3 shows that the first-order model has a relatively better fit.

### Analysis 2: scales reliability

Table 4 shows subscale descriptive statistics and the reliability of each factor and the full scale. The Cronbach alpha reliability for the five QUEVA-G subscales and the full scale indicated high to excellent internal consistency (alphas from 0.82 to 0.94).

**Table 4:** Descriptive statistics and reliability coefficients of the QUEVA-G.

Subscale	Alpha	Number of items	*M*	*SD*
**Parent–Assessor Relationship and Collaboration (PARC)** The assessor was genuinely interested in helping us. I felt the assessor respected me. I was informed about each step of the assessment. I liked the assessor. I trusted the assessor. I felt that my opinion was valued. The assessor really listened to me.	0.94	7	27.50	7.326
**New Understanding of the Child (NUC)** I have lots of new ideas about how to parent my child. I learned a tremendous amount about my child from this assessment. I am better able to communicate with my child. Now I know what to expect from my child. I understand my child so much better now	0.89	5	17.40	4.688
**Child–Assessor Relationship (CAR)** My child felt comfortable with the assessor. My child never really warmed up to the assessor (R). My child and the assessor really connected well. My child did not like the assessor (R).	0.84	4	15.50	4.035
**Systemic Awareness (SA)** My child’s problems are partly caused by other struggles in our family. Many of my child’s difficulties have to do with our family. The assessment revealed how family members play a role in my child’s problems. I now see how our family’s problems affect my child.	0.85	4	7.47	3.918
**Negative Feelings (NF)** The assessment made me feel ashamed. I felt blamed for my child’s problems. The assessment made me feel like a bad parent. I felt judged by the assessor.	0.82	4	16.98	3.765
**Questionario sull’Esperienza della Valutazione dei Genitori (QUEVA-G)**	0.91	24	84.84	16.29

*n* = 185. (R), reverse scored.

### Analysis 3: relationship of QUEVA-G subscales to overall satisfaction

Correlation analysis showed a strong positive correlation (*r* = 0.83) between the QUEVA-G total score and the CSQ-8 score, which represents parents’ General Satisfaction with the received service. This suggests that the parents’ satisfaction measured by the CSQ-8 has a substantial overlap with the one measured by QUEVA-G items, thus indicating a strong convergent construct validity. Correlations computed between the QUEVA-G subscales and the CSQ-8 total score showed statistically significant coefficients for every QUEVA-G subscale, except for the Systemic Awareness factor (Table 5).

**Table 5:** Correlation coefficients between CSQ-8 and QUEVA-G results.

	QUEVA-G					
CSQ-8 total score	Subscale					Total score
	PARC	NUC	CAR	SA	NF_(R)	
	0.865**	0.656**	0.636**	0.030	0.533**	0.832**

*n* = 177. (R), reverse scored.**p < 0.01.

Specifically, results showed that the CSQ-8 total score is strongly and positively correlated with the PARC subscale (*r* = 0.86). This suggests that parental General Satisfaction measured by the CSQ-8 is strongly associated with the quality of the relationship and the degree of collaboration established between parents and the assessor. Furthermore, strong positive correlations were also found between the CSQ-8 total score and the New Understanding of the Child subscale (*r* = 0.66), the Child–Assessor Relationship one (*r* = 0.64), and the reversed “Negative Feelings” factor (*r* = 0.53). This indicates that parental satisfaction is positively correlated with the possibility of achieving a greater understanding of the child, the quality of the relationship between the child and the assessor, and the absence of negative feelings during the assessment.

**Figure 3:**
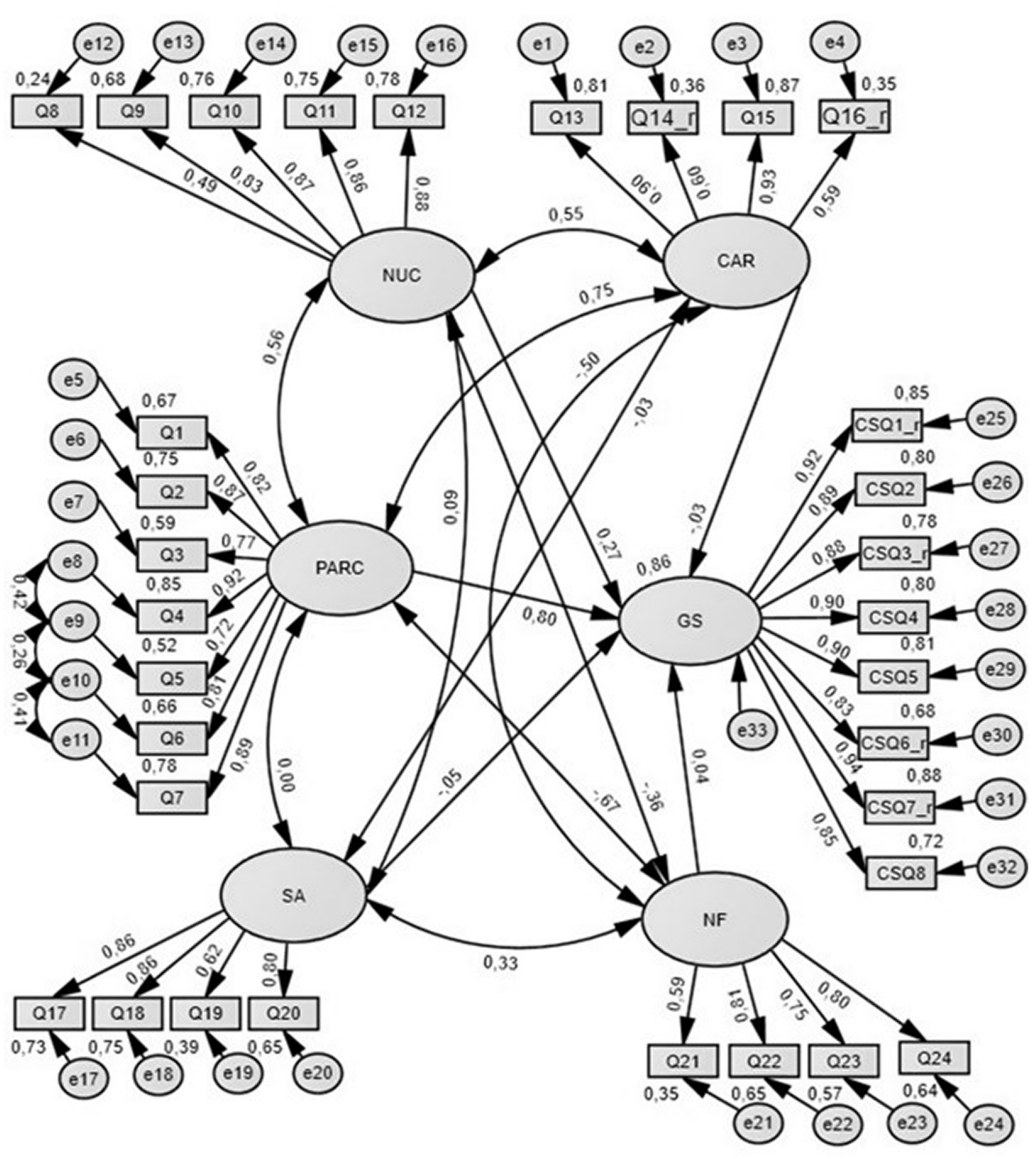
Effect of the QUEVA-G on General Satisfaction.

In addition, SEM was used to show the influence of each QUEVA-G subscale on General Satisfaction; in particular, we tested the fit and the paths among variables in a first-order model. In this configuration, we assumed that each of the five correlated factors could have a significant effect on the latent variable given by the CSQ-8 items called General Satisfaction. Modification indices suggested allowing the covariance of the error terms for the same items as the CFA (Figure 3).

Although *X^2^* for this model was statistically significant, all other fit indices suggested a good fit of the model to the data (Table 6). As shown in Table 7, the path analysis of our model suggested that the Parent-Assessor Relationship and Collaboration subscale (PARC) had the stronger significant direct effect on General Satisfaction (*β* = 0.802). Also, the New Understanding of Child subscale had a significant direct effect on GS (β = 0.266) even if weaker than PARC. The other QUEVA-G’s subscales, such as CAR (*β* = −0.033), SA (*β* = −0.054), and NF (*β* = 0.037), did not show a statistically significant effect.

**Table 6:** Model fit for the effect of the QUEVA-G on general satisfaction.

Model	*X^2^*	*df*	*p*	X^2^/*df*	TLI	CFI	RMSEA	95% CI for RMSEA	SRMR	AIC
First-order model	657.308	446	0.000	1.474	0.953	0.958	0.052	[0.043; 0.060]	0.0596	821.308

*X^2^*, Chi-square; df, degrees of freedom; *p, p* value; *X^2^*/*df*, discrepancy index; CFI, comparative fit index; RMSEA, root mean square of approximation; SRMR, standardized root mean square residual; TLI, Tucker-Lewis index; AIC, Akaike information criterion.

**Table 7:** Estimates of direct effects of the QUEVA-G on general satisfaction.

Subscale	Direct effect on general satisfaction	*p*
CAR	−0.033	0.601
**NUC**	**0.266**	**0.000**
**PARC**	**0.802**	**0.000**
SA	−0.054	0.231
NF	0.037	0.580

*n* = 177.

### Analysis 4: differences in parent experiences of psychological assessments

The MANOVA did not show any significant effect of children’s (Table 8) gender on the QUEVA-G results.

**Table 8:** Main effect of child’s gender on QUEVA-G results.

Subscale	*F* (*df*)	*p*	ƞ^2^	Gender of the child	*n*	*M*	*SD*
PARC	*F* (1;183) = 0.250	0.618	0.001	Male	127	3.902	1.013
				Female	58	3.985	1.123
NUC	*F* (1;183) = 0.091	0.763	0.000	Male	127	3.465	0.917
				Female	58	3.510	0.989
CAR	*F* (1;183) = 0.120	0.729	0.001	Male	127	3.892	0.988
				Female	58	3.836	1.061
SA	*F* (1;183) = 0.012	0.913	0.000	Male	127	1.862	1.020
				Female	58	1.879	0.891
NF_(R)	*F* (1;183) = 0.000	0.992	0.000	Male	127	4.244	0.960
				Female	58	4.246	0.907
Total Score	*F* (1;183) = 0.032	0.858	0.000	Male	127	17.36	3.052
				Female	58	17.46	3.550

*n* = 185. (R), reverse scored.

On the contrary, the administration of the QUEVA-G online led to statistically significant lower ratings for PARC (online *M* = 3.758; *SD* = 1.074; in person *M* = 4.657; *SD* = 0.443), NUC (online *M* = 3.385; *SD* = 0.968; in person *M* = 3.883; *SD* = 0.664), CAR (online *M* = 3.768; *SD* = 1.038; in person *M* = 4.329; *SD* = 0.722), NF (online *M* = 4.155; *SD* = 0.976; in person *M* = 4.629; *SD* = 0.654), and for the total score (online *M* = 16.92; *SD* = 3.282; in person *M* = 19.41; *SD* = 1.817) (Table 9). Effect sizes turned out to be small for NUC (η^2^ = 0.043), CAR (η^2^ = 0.048), and NF (η^2^ = 0.039), while for the total score and PARC, they were, respectively, medium (η^2^ = 0.093) and large (η^2^ = 0.114).

**Table 9:** Main effect of the format of administration on QUEVA-G results.

Subscale	*F* (*df*)	*p*	ƞ^2^	Format of administration	*n*	*M*	*SD*
**PARC**	***F* (1;183) = 23.501**	**0.000**	**0.114**	**Online**	**150**	**3.758**	**1.074**
				**Paper form**	**35**	**4.657**	**0.443**
**NUC**	***F* (1;183) = 8.310**	**0.004**	**0.043**	**Online**	**150**	**3.385**	**0.968**
				**Paper form**	**35**	**3.883**	**0.664**
**CAR**	***F* (1;183) = 9.139**	**0.003**	**0.048**	**Online**	**150**	**3.768**	**1.038**
				**Paper form**	**35**	**4.329**	**0.722**
SA	*F* (1;183) = 0.098	0.755	0.001	Online	150	1.857	1.004
				Paper form	35	1.914	0.876
**NF_(R)**	***F* (1;183) = 7.436**	**0.007**	**0.039**	**Online**	**150**	**4.155**	**0.976**
				**Paper form**	**35**	**4.629**	**0.654**
**Total Score**	***F* (1;183) = 18.723**	**0.000**	**0.093**	**Online**	**150**	**16.92**	**3.282**
				**Paper form**	**35**	**19.41**	**1.817**

*n* = 185. (R), reverse scored.

Furthermore, when parents participated in assessments that dealt with emotional and behavioral issues (*M* = 2.300; *SD* = 1.303), compared with cognitive and neurodevelopmental issues (*M* = 1.685; *SD* = 0.834), their ratings of SA were significantly higher. Additionally, participants who experienced mixed (*M* = 3.725; *SD* = 1.243) or emotional and behavioral assessments (*M* = 3.650; *SD* = 1.145) scored lower ratings of NF compared with cognitive and neurodevelopmental evaluations (*M* = 4.398; *SD* = 0.800). SA’s effect size was small (η^2^ = 0.053), while NF’s effect size was medium (η^2^ = 0.100; Table 10).

**Table 10:** Main effect of the type of assessment on QUEVA-G results.

Subscale	*F (df)*	*p*	η^2^	Type of assessment	Comparison	*p*	*n*	*M*	*SD*
PARC	*F* (2;148) = 0.972	0.381	0.013	Cognitive	Emotional	0.615	116	3.947	1.028
					Mixed	0.490			
				Emotional	Cognitive	0.615	15	3.676	1.029
					Mixed	0.998			
				Mixed	Cognitive	0.490	20	3.657	1.175
					Emotional	0.998			
NUC	*F* (2;148) = 1.563	0.213	0.021	Cognitive	Emotional	0.210	116	3.465	0.904
					Mixed	0.959			
				Emotional	Cognitive	0.210	15	3.013	1.205
					Mixed	0.268			
				Mixed	Cognitive	0.959	20	3.429	1.155
					Emotional	0.268			
CAR	*F* (2;148) = 2.264	0.108	0.030	Cognitive	Emotional	0.137	116	3.888	0.990
					Mixed	0.477			
				Emotional	Cognitive	0.137	15	3.350	1.194
					Mixed	0.754			
				Mixed	Cognitive	0.477	20	3.600	1.077
					Emotional	0.754			
**SA**	***F* (2;148) = 4.107**	**0.018**	**0.053**	**Cognitive**	**Emotional**	**0.042**	**116**	**1.685**	**0.834**
					Mixed	0.170			
				**Emotional**	**Cognitive**	**0.042**	**15**	**2.300**	**1.303**
					Mixed	0.777			
				Mixed	Cognitive	0.170	20	2.087	1.052
					Emotional	0.777			
**NF_(R)**	***F* (2;148) = 8.199**	**0.000**	**0.100**	**Cognitive**	**Emotional**	**0.008**	**116**	**4.398**	**0.800**
					**Mixed**	**0.007**			
				**Emotional**	**Cognitive**	**0.008**	**15**	**3.650**	**1.145**
					Mixed	0.968			
				**Mixed**	**Cognitive**	**0.007**	**20**	**3.725**	**1.243**
					Emotional	0.968			
Total Score	*F* (2;148) = 1.595	0.206	0.021	Cognitive	Emotional	0.253	116	17.38	3.001
					Mixed	0.842			
				Emotional	Cognitive	0.253	15	16.00	4.110
					Mixed	0.570			
				Mixed	Cognitive	0.842	20	16.60	3.565
					Emotional	0.570			

*n* = 151. (R), reverse scored.

Finally, assessments of older children were experienced more positively by parents, in PARC (4–11 years-old *M* = 3.760; *SD* = 1.095; 12–18 years-old *M* = 4.254; *SD* = 0.864), NUC (4–11 years-old *M* = 3.381; *SD* = 0.963; 12–18 years-old *M* = 3.670; *SD* = 0.861), SA (4–11 years-old *M* = 1.766; *SD* = 0.924; 12–18 years-old *M* = 2.063; *SD* = 1.059), and the total score (4–11 years-old *M* = 16.97; *SD* = 3.312; 12–18 years-old *M* = 18.21; *SD* = 2.842). All of these effect sizes were small (Table 11).

**Table 11:** Main effect of child’s age on QUEVA-G results.

Subscale	*F (df)*	*p*	η^2^	Age range	*n*	*M*	*SD*
**PARC**	***F* (1;183) = 9.693**	**0.002**	**0.050**	**4–11**	**122**	**3.760**	**1.095**
				**12–18**	**63**	**4.254**	**0.864**
**NUC**	***F* (1;183) = 4.004**	**0.047**	**0.021**	**4–11**	**122**	**3.381**	**0.963**
				**12–18**	**63**	**3.670**	**0.861**
CAR	*F* (1;183) = 0.275	0.601	0.002	4–11	122	3.846	0.987
				12–18	63	3.929	1.055
**SA**	***F* (1;183) = 3.883**	**0.050**	**0.021**	**4–11**	**122**	**1.766**	**0.924**
				**12–18**	**63**	**2.063**	**1.059**
NF_(R)	*F* (1;183) = 0.302	0.583	0.002	4–11	122	4.217	0.977
				12–18	63	4.298	0.873
**Total Score**	***F* (1;183) = 6.419**	**0.012**	**0.034**	**4–11**	**122**	**16.97**	**3.312**
				**12–18**	**63**	**18.21**	**2.842**

*n* = 185. (R), reverse scored. PARC, Parent–Assessor Relationship and Collaboration; CAR, Child–Assessor Relationship; NUC, New Understanding of the Child; SA, Systemic Awareness; NF, Negative Feelings; GS, General Satisfaction.

These results suggest that, in our study, parents completing the QUEVA-G online had more negative experiences during their children’s assessment than those completing it in person right after its conclusion. Whether this finding suggests that parents participating in self-help groups online might have actually experienced fewer fulfilling assessments or if the administration format might have enhanced a social desirability response set in parents completing the QUEVA-G in person is still unclear. The relatively better experience of parents whose children were older at the time of the assessment suggests that the child’s age may also play a role in the overall experience of their assessment.

## Discussion

Our study aimed to describe the psychometric properties of the Parent Experience of Assessment Scale (PEAS; Austin, 2011), translated into Italian, in an Italian sample of parents. We found that the QUEVA-G is a five-factor questionnaire with a good fit to the data, excellent reliability, and predictive validity for parents’ general satisfaction.

Our findings suggest that establishing a positive and collaborative relationship with parents, facilitating parents’ development of a new and more respectful understanding of the child, allowing a more positive perception of the parent–child relationship, and providing a positive emotional experience to all participants are very highly correlated processes. Of note, parents’ greater systemic awareness is correlated with their negative feelings about the assessment, suggesting that when parents acknowledge their own responsibility for their child’s difficulties, they are likely to experience negative feelings, such as guilt and shame. Future studies should try to discern whether this result is inherent to parents’ experience of their child’s assessments or if it is related to the specific ways assessments are performed.

SEM findings suggest that parental satisfaction with their child’s assessment is mostly predicted by parents’ positive and collaborative relationship with the assessor. This result is consistent with the research hypotheses that Austin et al. (2016) initially wanted to demonstrate but did not, since the PARC’s effect in their model is extremely weak and negative, as well as in their second-order model, it has only an indirect but moderate effect through CAR and NUC. In addition, our analysis suggests that the assessor’s ability to establish a positive and collaborative relationship with parents is not sufficient to enhance parents’ satisfaction. On the other hand, results suggest that higher parent satisfaction was correlated with a better understanding of their child’s problems.

Our analyses highlight the existence of some variables that can affect parents’ perception of their child’s assessment and, therefore, their level of satisfaction. First, differences in QUEVA-G scores emerged regarding the type of assessment received. Specifically, parents whose children received an assessment for emotional and behavioral distress achieved higher levels of systemic awareness than those whose children received cognitive and neurodevelopmental assessments. In addition, the former experienced more negative feelings than the latter. This perception is consistent with the above-mentioned statement that certain parents may feel uncomfortable acknowledging their role in their child’s difficulties. This finding suggests that it would be useful for clinicians to help parents overcome their negative emotions of guilt and shame and promote compassionate and beneficial solutions for the entire family. This is consistent with the following two goals that Therapeutic Assessment practitioners strive to achieve: (1) to improve parental systemic awareness about their child’s problems and (2) to empower parents to feel more self-assured and capable of finding solutions (Finn, 2007).

Finally, other differences were found relating to the child’s age: parents of adolescents (12–18 years) achieved higher scores than parents of younger children (4–11); therefore, it seems that the former were globally more satisfied.

### Limitations and future directions

Although the sample size was above the minimum 100 cases recommended for CFA (MacCallum et al., 1996, 1999), a larger sample size would have provided even stronger data in terms of the fit of models.

There was some variability in the data collection procedures (paper form or electronic form). Parents who completed QUEVA-G in the paper form at the clinics reported more global satisfaction than those who completed it online. Specifically, the analysis revealed that the latter reported a weaker relationship with the evaluator, a lower-quality perception of the evaluator–child relationship, a worse understanding of the child, and more negative feelings. It could be speculated that this result may be due to a general distrust toward the assessors. Future studies should be carried out with more homogenous and/or controlled samples to capture the differences between groups regarding satisfaction with the service received (such as comparing public and private services).

Furthermore, given that the majority of our sample comprised female participants, it would be worthwhile to consider administering the QUEVA-G to fathers as well, as previous research has shown that respondents’ gender can influence their experience of clinical interventions (Cooper et al., 2019).

The fit of the QUEVA-G to the data was good. However, based on the modification indices suggested by AMOS, we allowed correlating error terms for three items (4 and 5, 5 and 6, and 6 and 7), implying that there could be an additional construct or unexplored thematic area influencing these items. The correlation among the error terms may reflect the presence of a residual variance unaccounted for by the five factors considered in the model. Moreover, items might be formulated ambiguously, thus needing a revision. Further research should focus on these items.

Future studies should also address whether the QUEVA-G maps all the possible areas of parental experience using a qualitative approach. Indeed, QUEVA-G seems to be more focused on what happens in the assessment room in terms of relationships, effects, and feelings, while it could be further investigated, for example, what happens outside (e.g., relationships with other services; Aschieri et al., 2023).

## Conclusion

This study represents an initial effort to address the gap concerning measurement instruments for parental satisfaction with child assessments. While Larsen et al. (1979) previously considered parental satisfaction as a monofactorial construct, there is now significant evidence highlighting its multidimensional nature (Lewis, 1994). Compared to commonly used single-factor satisfaction measures, the QUEVA-G enables more precise reporting of various facets of parents’ experiences during their child’s psychological assessment, offering valuable insights for clinical practice and quality assurance programs.

Finally, the present study provides evidence for supporting the theoretical hypotheses of Therapeutic Assessment (TA), for instance, by demonstrating the crucial role of the PARC subscale compared to the other factors. Indeed, the present study highlights the great importance of the family-assessor relationship in parent satisfaction with the assessment process, which is consistent with prior research findings on this theme (Pascoe, 1983; Sheppard, 1993; Lewis, 1994), and with research stressing the need of actively involve families in the delivery of mental health services (Bogenschneider et al., 2012; Carrà, 2018).

## Data availability statement

The raw data supporting the conclusions of this article will be made available by the authors, without undue reservation.

## Ethics statement

The studies involving humans were approved by the Catholic University of the Sacred Heart. Number of institutional review board approval: 42–23. The studies were conducted in accordance with the local legislation and institutional requirements. The participants provided their written informed consent to participate in this study. Written informed consent was obtained from the individual(s) for the publication of any potentially identifiable images or data included in this article.

## Author contributions

FA: Methodology, Conceptualization, Funding acquisition, Resources, Supervision, Validation, Writing – review & editing. SB: Methodology, Data curation, Formal analysis, Investigation, Software, Writing – original draft. AC: Data curation, Supervision, Writing – review & editing. GC: Data curation, Formal analysis, Investigation, Methodology, Software, Writing – original draft.
