# Supplementary material for: Psychometric properties of the Italian version of the Parent Experience of Assessment Scale
Source: Front Psychol. 2024 Feb 1;14:1271713. doi: 10.3389/fpsyg.2023.1271713 (PMC10868526; doi:10.3389/fpsyg.2023.1271713)
Supplement: Supplementary file 1 [file Data_Sheet_1.docx]

**Appendix A**

*Questionario sull’Esperienza di Valutazione dei Genitori (QUEVA-G).*

**Questionario sull’Esperienza di Valutazione – Genitori (QUEVA-G)**

| Questo questionario contiene domande circa i Suoi pensieri e i Suoi sentimenti a proposito della valutazione psicologica e neuropsicologica di Suo/a figlio/a. Per favore, legga ciascuna delle seguenti affermazioni con attenzione. Una volta deciso il grado di accordo o disaccordo con ciascuna affermazione, segni la risposta che meglio lo rappresenta. Risponda nel modo più onesto e accurato possibile. Per favore, non salti nessuna riga e segni una sola risposta per ciascuna affermazione | | | | | |
| --- | --- | --- | --- | --- | --- |
|  | | | | | |
| **Utilizzare questa scala per valutare ciascuna affermazione:** | **Per niente d’accordo** | **Abbastanza in disaccordo** | **Neutro** | **Abbastanza**  **d’accordo** | **Molto d’accordo** |
| 1. L’operatore/trice era davvero interessato/a ad aiutarci. | 1 | 2 | 3 | 4 | 5 |
| 2. Ho sentito che l’operatore/trice mi rispettava. | 1 | 2 | 3 | 4 | 5 |
| 3. Sono stato/a informato/a su ciascun passaggio della valutazione. | 1 | 2 | 3 | 4 | 5 |
| 4. Mi è piaciuto/a l’operatore/trice. | 1 | 2 | 3 | 4 | 5 |
| 5. Mi sono fidato/a dell’operatore/trice. | 1 | 2 | 3 | 4 | 5 |
| 6. Ho sentito che la mia opinione era importante. | 1 | 2 | 3 | 4 | 5 |
| 7. L’operatore/trice mi ha davvero ascoltato/a. | 1 | 2 | 3 | 4 | 5 |
| 8. Ho molte idee nuove su come educare mio/a figlio/a. | 1 | 2 | 3 | 4 | 5 |
| 9. Ho imparato moltissime cose su mio/a figlio/a attraverso la valutazione. | 1 | 2 | 3 | 4 | 5 |
| 10.Sono più capace di comunicare con mio/a figlio/a. | 1 | 2 | 3 | 4 | 5 |
| 11.Ora so cosa aspettarmi da mio/a figlio/a. | 1 | 2 | 3 | 4 | 5 |
| 12.Ora capisco molto meglio mio/a figlio/a. | 1 | 2 | 3 | 4 | 5 |
| 13.Mio/a figlio/a era a suo agio con l’operatore/trice. | 1 | 2 | 3 | 4 | 5 |
| **Utilizzare questa scala per valutare ciascuna affermazione:** | **Per niente d’accordo** | **Abbastanza in disaccordo** | **Neutro** | **Abbastanza**  **d’accordo** | **Molto d’accordo** |
| 14.Mio/a figlio/a non si è mai davvero coinvolto/a con l’operatore/trice. | 1 | 2 | 3 | 4 | 5 |
| 15.Mio/a figlio/a e l’operatore/trice erano davvero in sintonia. | 1 | 2 | 3 | 4 | 5 |
| 16.A mio/a figlio/a non piaceva l’operatore/trice. | 1 | 2 | 3 | 4 | 5 |
| 17.I problemi di mio/a figlio/a sono in parte causati da altri conflitti nella mia famiglia. | 1 | 2 | 3 | 4 | 5 |
| 18.Molte delle difficoltà di mio/a figlio/a hanno a che fare con la nostra famiglia. | 1 | 2 | 3 | 4 | 5 |
| 19.La valutazione ha messo in luce il ruolo dei famigliari nei problemi di mio/a figlio/a. | 1 | 2 | 3 | 4 | 5 |
| 20.Ora vedo come i problemi della nostra famiglia influenzano mio/a figlio/a. | 1 | 2 | 3 | 4 | 5 |
| 21.La valutazione mi ha fatto provare vergogna. | 1 | 2 | 3 | 4 | 5 |
| 22.Mi sono sentito/a criticato/a per via dei problemi di mio/a figlio/a. | 1 | 2 | 3 | 4 | 5 |
| 23.La valutazione mi ha fatto sentire un cattivo genitore. | 1 | 2 | 3 | 4 | 5 |
| 24.Mi sono sentito/a giudicato/a dall’operatore/trice. | 1 | 2 | 3 | 4 | 5 |
